# Can L‐glutamine augmented heat shock protein 70 expression prevent exercise‐induced exertional heat stroke and sudden cardiac death?

**DOI:** 10.1111/cns.13217

**Published:** 2019-08-30

**Authors:** Yang Zhang, Phillip A. Bishop

**Affiliations:** ^1^ Faculty for Sport and Physical Education University of Montenegro Podgorica Montenegro; ^2^ Department of Kinesiology University of Alabama Tuscaloosa AL USA

Dear Editor‐in‐Chief,

Glutamine has been shown to protect cells, tissues, and whole organisms from stress and injury.^1^ This protection has largely been thought to be related to augmented expression of 70‐kDa heat shock protein (Hsp70). Hsp70 is expressed in normal cells and can be enhanced by environmental stresses (eg, heat, and hypoxia) and pathophysiological states (eg, inflammation, ischemia). Hsp70 acts in chaperoning the folding, aggregation, or degradation of other proteins and functions in maintaining the metabolic and structural integrity of the cell, as a protective response to external stresses. Much work has been done in recent years to explore the therapeutic role of Hsp70 expression in several types of diseases, including Alzheimer's disease, heart disease, stroke, and cancer. Briefly, Hsp70 exerts the cytoprotective properties through its capacity of tissue protection, preservation of tissue metabolic function in stress states, and anti‐inflammatory and antioxidant regulation.^2^ Despite well‐studied mechanisms related to the stress response in clinical settings, the investigation of Hsp70 applications in health and disease remains limited because the expression is usually subject to external stimuli. Recent advances using pharmacologic supplementation of glutamine^3^ shed new light in this regard and may offer a viable option for potentiating Hsp70 response prior to, and following, stressors (eg, hyperthermia, and cardiac strain) in humans in a controlled and efficient manner.

## 1 MODE OF ACTION

Of great interests regarding the protective effects of the Hsp70 is its role in the development of heatstroke and myocardial ischemia. First, Hsp70 expression functions as cryoprotectants during hyperthermia and heatstroke.^4^ Hsp70 is well known to provide cells with elevated thermal resistance, which is critical for the survival of most living organisms that are exposed to extreme temperatures. During heat stress, Hsp70 provides cells with time to repair damage and prevents necrosis. It has been suggested that the relative resistance to heatstroke in the canine heatstroke model is related to the upregulation of Hsp70.^4^ Its protective role in the pathophysiology of hyperthermia and heatstroke is unequivocal.

Second, Hsp70 overexpression protects against lethal injuries such as myocardial infarction. Data have shown that an infarcted heart has a lower production capacity of Hsp70 and consequently, this reduction in the expression of Hsp70 leads to the further decrease in contractile function during subsequent development of myocardial infarction.^2^ Conversely, Hsp70 overexpression modulates the process of intracellular repair by reducing heart necrosis in coronary heart disease and thereby provides a significant reduction in the infarcted area as well as improved postischemic contractile recovery. Current evidences suggest that Hsp70 exhibits a protective effect in heart and brain tissue against repeated ischemic injury.

Third, glutamine, in part dependent on Hsp70 expression, is a powerful anti‐oxidant, antiinflammatory, and pharmacologic agent that regulates immune response at a number of different levels.^1^ Hsp70‐mediated anti‐inflammation action has been noted to attenuate nuclear factor NF‐κB and inhibitor factor IκBα signaling pathways. Hsp70 also interacts with the activation of the stress kinase pathway. This inhibitory effect could attenuate tumor necrosis factor‐α, interleukin‐6, and interleukin‐18 expression after sepsis, thereby fulfilling various cellular‐level anti‐inflammatory and stress‐induced functions. Remarkably, inflammation plays a pivotal role in the development of cardiovascular disease. In this regard, the mechanisms of Hsp70 expression in antioxidation and anti‐inflammation underscore an important implication for a therapeutic role of L‐glutamine in cardiovascular disease.

Given the importance of Hsp70 in a range of cytoprotective and immunomodulatory cascade reactions, insightful examination of the physiological and immunological role of Hsp70 is warranted, as this will facilitate the development of optimal approaches for reducing morbidity and mortality in heat susceptible soldiers, firefighters, and athletes. In this regard, the therapeutic potential of modulating Hsp70 expression via L‐glutamine supplementation is of particular interest.

## 2 TARGETED PREVENTION

Extreme high temperature is one of the most important environmental stresses experienced by soldiers, firefighters, and athletes. Prolonged physical exertion in the heat could cause continuous rise in core temperature, which could cause exertional heat illness, including life‐threatening heatstroke. To date, exertional heatstroke is still a persistent cause of morbidity and mortality among susceptible individuals who obligatorily undertake intense exercise in the heat. A cohort study of the US Army tracking incident rates of heat illness hospitalizations and death reported an eightfold increase in heatstroke hospitalization rates from 1980 to 2002.^5^ Moreover, it is crucial to recognize that physical exertion in the heat, combined with protective clothing and equipment required in combat, labor or athletics can lead to dangerous heat strain even in mild environments.

A particularly relevant note is that firefighters regularly perform fire simulation tasks or are deployed to daily exertional training. A wealth of literature of experimental studies in cohorts of career firefighters suggests that myocardial inflammation could occur following a short bout of fire simulation training (eg, 20 minutes),^6^ highlighting that potentially adverse cardiovascular manifestations (eg, thrombogenicity, platelet activation, and impaired vasomotor endothelial function) may be sustained on a daily basis throughout their career. Not surprisingly, it is well known that firefighting is associated with acute myocardial ischemia and sudden cardiac death, which is the leading cause of duty‐related fatalities among US firefighters.^6^

In general, soldiers, firefighters, and athletes are at increased risks of exertional heatstroke and sudden cardiac death due to obligations of strenuous exercise under high ambient temperatures. Novel strategies supplementing the current methods should be explored as potential preventive measures and post‐incident medical care against these lethal injuries.

## 3 FUTURE OF L‐GLUTAMINE AUGMENTED HSP70 EXPRESSION

At present, there are few if any preventive measures against sudden cardiac death resulting from exercise‐heat stress among firefighters and athletes. Should exposure to heat and/or physical exertion lead to accelerated immune and inflammatory changes among these cohorts, preventive measures are urgently needed to minimize such adverse changes. However, an agent that is truly effective and without harm is yet to be established.^7^ Prior works from clinical settings and animal models have identified that myocardial tolerance against ischemic injury can be improved as a result of prior induction or overexpression of the Hsp70.^2^ The involvement of L‐glutamine could be crucial because its antioxidant and anti‐inflammatory effects may be an useful and practical solution for reducing firefighting‐induced myocardial inflammation. Moreover, altered immune and inflammatory responses during and beyond the immediate heat exposure increase the risk of an acute coronary event,^6^ for which increased circulating levels of Hsp70 via L‐glutamine supplementation could activate innate immune mechanisms and promote myocardial survival^2^ and thereby may provide a new pharmacological measure to reduce subsequent risks of cardiac events.

With regard to exertional heatstroke, heat acclimatization/acclimation and hydration have been recommended as preventive measures but the prevalence of illness persists. Post‐incident medical care of exertional heatstroke includes cold water immersion and intravenous administration of fluids. It is important to highlight that fatal cases of exertional heatstroke show widespread muscle necrosis,^8^ demonstrating heat shock‐induced cell death is an unfortunate comorbidity in this medical condition. Hsp70 is well known to elevate thermotolerance of cells to protect vital organs from the damaging effects of hyperthermia. Should basal Hsp70 level be up‐regulated and the response rate following heat stress be accelerated, this molecular chaperone could attenuate heatstroke‐induced inflammation, potentiate innate immunity, and reduce cellular stress or necrosis.^4^, ^9^ Primarily, L‐glutamine may be implemented as a preventive or therapeutic agent to reduce the increasing frequency of life‐threatening exertional heatstroke among soldiers and athletes.^5^, ^10^ Additionally, short‐term use of L‐glutamine may be a beneficial prevention and management strategy for unacclimated individuals to reduce heat illness during the initial days of exercise in the heat. L‐glutamine, albeit its effect is yet to be established, could be especially valuable for soldiers because adequate heat acclimatization is often not possible before immediate deployment to hot climatic zones. This effect, together with the myocardial protection against ischemia, may play a key role in the prevention of exertional heatstroke and sudden cardiac death.

To date, L‐glutamine has not entered clinical trials as a targeted preventive treatment for exercise‐induced exertional heatstroke or sudden cardiac death. Accordingly, the study by Luo and colleagues^3^ offers translational insights for a larger realm of human research and clinical applications. Primarily, the clinical value of L‐glutamine supplementation in response to acute exercise‐heat stress should be assessed. Specially, research is needed to investigate its pharmacokinetics and efficacy for guiding preventative practice before exercise in the heat, or ad‐hoc medical treatment after exertional heatstroke or acute myocardial ischemia. A recent work has demonstrated Hsp70 overexpression in firefighters as a result of oral glutamine ingestion (0.15 g/kg/day)^11^; further research is warranted to determine the dose‐response relationship following acute and chronic supplementation. However, establishing the optimal dose of L‐glutamine as a preventive measure will probably be difficult due to ethical issues and individual variability in thermotolerance or cardiac risks.

Additionally, there are other unanswered questions about the practical utility of L‐glutamine. An important consideration that must be addressed is drug safety. Short‐term supplementation of glutamine appears to be safe; however, there are no clear data regarding the long‐term safety. Prior work has revealed that critically ill patients with multiorgan failure had paradoxically higher mortality rates when treated with a combination of IV and enteral glutamine (0.35 g/kg/day and 30 g/day, respectively).^12^ Notably, low glutamine levels have been detected in critically ill patients, which in turn completely inhibit Hsp70 expression thereby damaging monocytes during hyperthermia.^13^ Nevertheless, the overall efficacy of L‐glutamine supplementation in occupational and athletic settings most certainly warrants exploration.

L‐glutamine augmented Hsp70 expression is established science. Indeed, more than a decade ago it was reported that glutamine supplementation enhanced Hsp70 expression and improved survival following hyperthermia.^14^ The clinical benefit of L‐glutamine revealed by Luo and colleagues^3^ appears to be promising and opens up translationally unexamined research possibilities. Practically speaking, L‐glutamine can be prescribed in IV administration, and more conveniently, it can be taken orally and available in over‐the‐counter formulation. The functional role of L‐glutamine in exercise physiology and exercise immunology is currently in its infancy, thus, published primary data regarding the drug efficacy and safety will first be needed for L‐glutamine to gain common acceptance by the clinical community and practitioners. Taken collectively, we envision L‐glutamine may constitute the first pharmacological candidate to mitigate specific risks of medical complications for soldiers, firefighters, and athletes undergoing heat exposure.

## COMPETING INTERESTS

None declared.
